# Effective bridging strategies prior to infusion with tisagenlecleucel results in high response rates and long-term remission in relapsed/refractory large B-cell lymphoma: findings from a German monocentric study

**DOI:** 10.1007/s00432-024-05765-8

**Published:** 2024-05-01

**Authors:** Farina Eigendorff, Irina Filimonova, Sebastian Scholl, Anne Sayer-Klink, Silke Rummler, Christa Kunert, Klaus Pietschmann, Andrea Wittig, Andreas Hochhaus, Ulf Schnetzke

**Affiliations:** 1 Klinik Für Innere Medizin II, Abteilung für Hämatologie und Internistische Onkologie, Universitätsklinikum Jena, Am Klinikum 1, 07747 Jena, Germany; 2 Klinik für Strahlentherapie und Radioonkologie, Universitätsklinikum Jena, Jena, Germany; 3 Institut für Transfusionsmedizin, Universitätsklinikum Jena, Jena, Germany; 4 Comprehensive Cancer Center Central Germany (CCCG) Jena/Leipzig, Campus Jena, Jena, Germany; 5 Klinik und Poliklinik für Strahlentherapie und Radioonkologie, Universitätsklinikum Würzburg, Würzburg, Germany

**Keywords:** CAR-T, Large B-cell lymphoma, Tisagenlecleucel, Radiotherapy, Bridging

## Abstract

**Background:**

Incorporating chimeric antigen receptor (CAR)-T cell therapy into relapsed or refractory large B-cell lymphoma (rr LBCL) treatment algorithms has yielded remarkable response rates and durable remissions, yet a substantial portion of patients experience progression or relapse. Variations in outcomes across treatment centers may be attributed to different bridging strategies and remission statuses preceding CAR-T cell therapy.

**Patients:**

Twenty-nine consecutive adult patients receiving tisagenlecleucel (tisa-cel) for rr LBCL from December 2019 to February 2023 at Jena University Hospital were analyzed.

**Results:**

The median age was 63, with a median of 3 prior treatments. Twenty patients (69%) were refractory to any systemic therapy before CAR-T cell treatment. Following leukapheresis, 25 patients (86%) received bridging therapy with the majority undergoing chemotherapy (52%) or combined modality therapy (32%). Radiotherapy (RT) was part of the bridging strategy in 44%, with moderately hypofractionated involved site RT (30.0 Gy/2.5 Gy) being applied most frequently (64%). Post-CAR-T infusion, the objective response rate at 30 days was 83%, with 55% achieving complete response. Twelve-month progression-free (PFS) and overall survival (OS) were 60% and 74%, respectively, with a median follow up of 11.1 months for PFS and 17.9 months for OS. Factors significantly associated with PFS were chemotherapy sensitivity pre-leukapheresis and response to bridging.

**Conclusion:**

The study underscores the importance of minimal tumor burden at CAR-T initiation, emphasizing the need for suitable bridging regimens. The findings advocate for clinical trials and further real-world analyses to optimize CAR-T cell therapy outcomes by identifying the most effective bridging strategies.

**Supplementary Information:**

The online version contains supplementary material available at 10.1007/s00432-024-05765-8.

## Introduction

Over the past years, significant strides have been made in the field of immunotherapy, revolutionizing our approach to cancer treatment. Among the remarkable advancements in this domain, the introduction of Chimeric Antigen Receptor (CAR)-T cell therapy marked a paradigm shift in lymphoma therapy. Tisagenlecleucel (tisa-cel, Kymriah^©^) was the first CD19 directed CAR-T therapy to be approved for the use in children and young adults with relapsed or refractory (rr) acute lymphoblastic leukemia (ALL) by the U.S. Food and Drug Administration (FDA) in 2017 (Maude et al. 2018). In 2018 tisa-cel was approved both by the FDA and European Medicines Agency (EMA) for patients with rr large B-cell lymphoma (LBCL) who did not respond after two or more previous systemic treatment lines (Schuster et al. 2019, 2021). Two other CAR-T products, axicabtagene ciloleucel (axi-cel, Yescarta^©^) and lisocabtagene maraleucel (liso-cel, Breyanzi^©^) have been approved for rr LBCL (Neelapu et al. 2017; Abramson et al. 2020). Based on the results of the prospective randomized controlled phase 3 trials ZUMA-7 and TRANSFORM the FDA and EMA approved both axi-cel and liso-cel for patients with LBCL who experience refractory disease or relapse within 12 months of first-line treatment (Locke et al. 2022; Abramson et al. 2023). The phase 3 randomized controlled BELINDA trial investigating tisa-cel versus standard of care (SOC) high dose chemotherapy with autologous stem cell transplant (auto-HCT) did not show superiority of the CAR-T product (Bishop et al. 2022). Intrinsic differences in product efficacy might be partly responsible for those discrepancies but inter-trial differences should also be taken into account (Westin and Sehn 2022).

Despite these advances in the treatment landscape for patients with rr LBCL, more than half of patients subsequently experienced disease progression or relapse following CAR-T therapy. Tisa-cel was approved based on the JULIET trial for rr LBCL after two or more lines of therapy. The long-term follow-up for this trial with a median of 40.3 months, reported a median progression free survival (PFS) of 2.9 months, and a median overall survival (OS) of 11.1 months (Schuster et al. 2021). T-cell intrinsic and extrinsic mechanisms of treatment failure are under investigation and addressed in early phase clinical trials (Strati and Neelapu 2021; Shah and Fry 2019). Furthermore, the delay between T-cell collection and infusion can lead to further progression of the disease or entirely prevent patients from being treated. Recent reports highlight the importance of bridging strategies prior to CAR-T infusion (Roddie et al. 2023; Bhaskar et al. 2022; Pinnix et al. 2020; Amini et al. 2022). Bridging therapy has become a widely used tool to stabilize or even debulk disease within the interval between leukapheresis and CAR-T cell administration. In the JULIET trial, time from apheresis to infusion ranged from 30 to 92 days (Schuster et al. 2019). Within this trial 92% of enrolled patients received bridging therapy most often consisting of chemotherapy including gemcitabine, etoposide or cisplatin. Data from commercial use of CAR-T therapy suggest a benefit of successful bridging regarding long term outcome of rr LBCL patients following CAR-T cell infusion (Bethge et al. 2022). In contrast, other studies have demonstrated that use of bridging therapy is associated with higher rates of toxicity and poorer overall survival (Pinnix et al. 2020; Nastoupil et al. 2020). This is primarily attributed to the utilization of bridging therapy in patients with refractory and more aggressive disease, and as such the need for disease control with bridging therapy likely identifies patients with a worse prognosis. Recent studies have also highlighted the detrimental effects of high tumor burden preceding CAR-T infusion (Locke et al. 2022; Bailly et al. 2022). Evidence indicates that patients achieving partial or complete remission following bridging therapy before undergoing CAR-T therapy exhibit favorable long-term outcomes (Roddie et al. 2023; Bachy et al. 2022). Consequently, there is a growing trend towards adopting more aggressive and personalized strategies for reducing tumor size through bridging approaches.

Some studies have shed light on the role of radiotherapy as bridging therapy (bRT). Preliminary evidence suggests that the combination of CAR-T therapy with bRT may enhance therapeutic benefits, not only through local antineoplastic effects but also by local and possibly systemic immunomodulatory mechanisms (Roddie et al. 2023; Fan et al. 2023; Fang et al. 2021; Sim et al. 2019; Saifi et al. 2022).

This single-center study, focusing on bridging therapy and the incorporation of radiation, particularly in chemorefractory patients, delineates a cohort of 29 consecutive treated rr LBCL patients who received tisa-cel after two or more lines of therapy.

## Patients and methods

### Patient cohort and informed consent

A total of 29 consecutive adult patients receiving tisa-cel for rr LBCL from December 2019 to February 2023 at Jena University Hospital were analyzed. All patients received treatment in accordance with the European Medicine Agency (EMA) approval label, specifically after undergoing at least two prior lines of treatment. Patient data were recorded in the European Group for Blood and Marrow Transplantation (EBMT) database. This retrospective study received approval by the Ethical Committee of the University of Jena (reg 2020–1626-Reg) and was conducted in compliance with the Declaration of Helsinki.

### Patient treatments

All patients received lymphodepleting chemotherapy (LD) with fludarabine and cyclophosphamide from day-5 to day-3 (fludarabine: 25–30 mg/m^2^ and cyclophosphamide: 250–500 mg/m^2^). Patients received tisa-cel infusion in an inpatient setting. Bridging therapy was defined as any therapy received between leukapheresis and start of LD. Patients were administered a bridging therapy based on chemotherapy alone, a combination of radiation and immunotherapy/chemotherapy (combined modality treatment, CMT), radiation alone, or immunotherapy alone. The decision to implement RT as part of the bridging protocol was made as case-by-case decision after interdisciplinary tumor board discussion. Main reasons to indicate bRT were chemotherapy-refractory and bulky disease.

### Safety analyses

Cytokine release syndrome (CRS) and immune effector cell associated neurotoxicity syndrome (ICANS) were graded in accordance with the ASTCT grading criteria and treated according current recommendations (Hayden et al. 2022; Lee et al. 2019).

The definition of CAR-T cell-mediated hematological toxicity followed the criteria established by Rejeski et al. and adhered to clinical trial criteria (CTCAE V5.0) (Rejeski et al. 2021). Severe neutropenia was characterized by an absolute neutrophil count (ANC) < 500/µl, and prolonged neutropenia was defined as ANC < 1000/µl and/or G-CSF dependence lasting ≥ 21 days after CAR-T infusion and continuing for ≥ 21 days. Prolonged severe thrombocytopenia was identified as platelet counts < 20/nL and/or requiring transfusions measured ≥ 21 days after CAR-T infusion and continuing for ≥ 21 days. Prolonged severe anemia was described as hemoglobin < 8 g/dL and/or requiring transfusions measured ≥ 21 days after CAR-T infusion and continuing for ≥ 21 days. Recovery was determined as a self-sustaining ANC > 1/nl without G-CSF support, a stable platelet count > 20/nL, and hemoglobin > 8 g/dL without transfusion requirement, respectively. Information regarding hematological toxicity excluded patients who experienced cytopenia as a result of subsequent therapy in the event of relapsed disease or secondary malignancies like myelodysplastic neoplasm.

### Response assessment

Response both of bridging therapy and CAR-T cell therapy was assessed based on the Lugano 2014 criteria, utilizing (Bailly et al. 2022) fluoro-deoxyglucose positron emission tomography (FDG-PET) at pre-defined timepoints: prior to bridging, at the time of LD, 1 month, 3 and 9 months after CAR-T infusion and in case of clinical suspicion of relapse (Cheson et al. 2014).

For all survival endpoints, survival was calculated from the date of CAR-T infusion. The analyzed outcome parameters included overall response rate (ORR), complete response rate (CR), progression free survival (PFS) rate and overall survival (OS). PFS was defined from the date of CAR-T infusion to the date of first documented relapse, progressive disease, or death from any cause, whichever occurred first. OS was defined from the date of CAR-T infusion to the date of death from any cause or the date of last follow-up.

### Statistics

Probabilities of OS and PFS were estimated using Kaplan–Meier plots and log-rank tests to identify differences between groups. The event-free probabilities at 12 months with 95% CIs were determined based on the number of patients at risk. The level of significance was 0.05 for all tests. Descriptive statistics employed absolute frequencies and percentages for categorical variables and medians and ranges for continuous variables. Group differences were evaluated using chi-square tests and Mann–Whitney’s rank sum test. Analyses were performed by SPSS 29.0 (SPSS Inc., Chicago, IL, USA) and GraphPad Prism Software 9.1.2 (GraphPad Software La Jolla CA, USA).

## Results

### Baseline characteristics

Patients’ characteristics are outlined in Table 1. Median age was 63 years (range 34–74). Twelve patients (41.4%) were female. Approximately half of the patients (48%) had a high-intermediate or high international prognostic index (IPI) at start of LD. An Eastern Cooperative Oncology Group performance status (ECOG PS) of ≥ 2 was observed in 20.7% of the patients. Prior to leukapheresis, 24 patients (83%) had received three or more lines of systemic therapy, and prior autologous stem cell transplantation (auto-SCT) had been performed in 41% (n = 12) of patients. Table S1 presents the patient characteristics of those who received bridging therapy. Specifically, it delineates patients who received radiation therapy as part of bridging (bRT) and those who did not (non-RT bridging). Notably, among patients who received radiation therapy, 82% were refractory to previous chemotherapy. Among the non-radiation therapy patients, this percentage was 50%.

**Table 1:** Patient characteristics

Patients infused	29
Median age, years (range)	63 (34 – 74)
Female, n, (%)	12 (41.4)
Histology	
DLBCL n, (%)	29 (100)
tFL n, (%)	5 (17.2)
LDH @ LD	
LDH ≤ ULN, n (%)	20 (70)
LDH > ULN, n (%)	9 (30)
IPI high-intermed. /high @ LD, n (%)	14 (48.3)
ECOG PS ≥ 2 @ LD, n (%)	6 (20.7)
Prior lines of therapies, median (range)	3 (2–4)
Prior auto-HCT, n (%)	12 (41.4)
≥ 3 treatment lines @ apheresis, n (%)	24 (82.8)
Interval leukapheresis to CAR-T infusion (days), median (range)	48 (28 – 203)
Bridging therapy, n, (%)	25 (86.2)
Response to last treatment prior apheresis	
CR/PR, n, (%)	11 (37.9)
SD/PD, n (%)	17 (58.6)
n.d., n (%)	1 (3.4)
Refractory to any line prior apheresis, n (%)	19 (65.5)

*DLBCL* diffuse large B-cell lymphoma; *tFL* transformed follicular lymphoma; *LDH* lactate dehydrogenase; *LD* lymphodepletion (lymphodepleting chemotherapy); *ULN* upper limit normal; *IPI* international prognostic index; *ECOG* eastern cooperative oncology group performance status; *auto-HCT* autologous hematopoietic cell transplantation; *CR* complete remission; partial remission; *SD* stable disease; *PD* progressive disease; *n.d.* no data

### Treatment characteristics including bridging strategies

A comprehensive description of the lines of chemotherapy prior to leukapheresis, including response, is provided in table S2. At the time of leukapheresis, more than half of the patients (n = 17, 59%) were refractory to the most recent treatment, and 66% (n = 19) exhibited refractoriness to any line of therapy before leukapheresis. Prior to LD, bridging therapy was administered in 85.2% (n = 25) of patients. Bridging modalities included classical chemoimmunotherapy (52%), immunotherapy (4%), RT only (12%) and combined radio- and immunotherapy (32%) (Table 2). A detailed description of the RT parameters is provided in Table 3.

**Table 2:** Bridging characteristics and efficacy

Patients bridged, n (%)	25 (86)
Chemotherapy	13 (52)
Immunotherapy only	1 (4)
Radiation only	3 (12)
CMT	8 (32)
Remission prior leukapheresis	
CR/PR	1 (4)
SD/PD	23 (92)
n.d	1 (4)
Response to bridging	
CR/PR	14 (56)
SD/PD	11 (44)
Conversion refractory to response by bridging	13 (52)

*CMT* combined modality treatment; *CR* complete remission; *PR* partial remission; *SD* stable disease; *PD* progressive disease; *n.d.* no data

**Table 3:** Treatment characteristics and response of patients with bridging radiotherapy

Patient #	Time between radiation and CAR-T infusion (days)	Total dose (Gy)	Dose per fraction (Gy)	Number of fractions	Pattern of disease (localized vs diffuse)	Target site	Irradiated total tumor volume (cc)	Response to bridging no/yes	Site of progression No progression inside RT field Outside RT field	Decrease in SUV post-bRT absolute value (% iSUV)	Concurrent systemic therapy
1	70	30.0	3.0	10	diffuse	Paraaortic Renal pelvis	268	Yes	No progression	− 49 (96)	Rituximab-Lenalidomide
2	14	30.0	2.5	12	localized	Paraaortic Common iliac Extern iliac	372	No	Outside RT Field	− 6.2 (71)	Rituximab-Bendamustine-Polatuzumab
3	13	36.0	2.0	18	localized	Orbita	8	Yes	No progression	*	none
4	8	30.0	2.5	12	diffuse	Paraaortic Mesenteric	1311	Yes	No progression	− 8 (74)	Lenalidomide- Venetoclax
5	17	30.0	2.5	12	localized	Paraaortic Common iliac Renal pelvis	2049	No	Outside RT field	− 21 (42)	Rituximab-Bendamustine-Polatuzumab
6	12	30.0	2.5	12	diffuse	Paraaortic Common iliac Renal pelvis Thigh	1304	No	Outside RT field	− 4 (26)	Rituximab-Bendamustine-Polatuzumab
7	12	30.0	2.5	12	localized	Lower leg	22	yes	No progression	− 9 (81)	none
8	13	30.0	2.5	12	diffuse	Lung	126	No	Outside RT field	− 11 (38)	Rituximab-ICE-Polatuzumab
9	14	30.0	2.5	12	localized	Mediastinal Lung	374	Yes	No progression	*	R-DHAOx
10	8	36.0	1.8	20 (bidaily)	diffuse	Extern iliac Inguinal	156	No	Outside RT field	0 (0)	Rituximab-Bendamustine-Polatuzumab
11	12	36.0	2.0	18	diffuse	Extern iliac Inguinal	319	No	Outside RT field	2 (31)	none

*Gy* Gray; *ICE* ifosfamide/carboplatin/etoposide, *DHAOx* dexamethasone/cytarabine/oxaliplatin; *SUV* standardized uptake value; *iSUV* initial SUV; *RT* radiotherapy

^*^no PET/CT available

### Radiotherapy as bridging modality

Modern state-of-the-art radiation techniques including intensity-modulated radiation therapy (IMRT), helical tomotherapy or Rapid Arc, volumetric modulated arc therapy (VMAT) with image-guidance (IGRT) were applied. The majority of patients (64%, 7/11) underwent moderately hypofractionated RT with single doses of 2.5 Gy, reaching a cumulative dose of 30.0 Gy within a time span of 2.5 weeks. The median EQD2α/β = 3 for all patients was 33.0 Gy (range, 33.0–36.0) and EQD2α/β = 10 31.3 Gy (range, 31.3 – 36.0).

The radiation field encompassed nodal involvement in 8 out of 11 patients (73%), extranodal manifestation in 3 out of 11 patients (27%), and both in one case (9%). In 7 out of 11 patients (64%), the planning target volume (PTV) comprised bulky lymph node lesion with a diameter of ≥ 5 cm.

In all 11 patients, involved site radiation was administered. The gross tumor volume (GTV) comprised solely the affected lymph node or extranodal involvement. When deemed appropriate, an internal target volume (ITV) was delineated. The safety margin was determined based on institutional positioning uncertainties and irradiation techniques (PTV, median = 1.0 cm, range, 0.2–2.0). The median total tumor volume (GTV) was 319 cc (range, 8–2049), while the median total PTV was 703 cc (range 17–3799) (Table 3). Within this cohort, the para-aortic region was the most frequently irradiated site (6/11, 55%) (Table S3). One patient had received prior irradiation at the same location (right lower leg).

### Response and toxicity to radiotherapy bridging

Prior to CAR-T infusion, of 25 patients who received bridging therapy, 11 (44%) received bRT. bRT was applied mainly in patients who were refractory to prior chemotherapy (10/11), had not received radiation therapy before (10/11), and did not have 3 or more sites of lymphoma disease at the time of leukapheresis (9/11). Bridging response prior to CAR-T infusion was assessed by PET-CT and response data are provided in Table 3. In 5 out of 11 patients (45%), a transition from refractory to responsive disease was attained through bRT. Sites of progression following bRT were observed to be located outside the radiation field in all patients. Importantly, a clinically significant reduction in standardized uptake value (SUV) was achieved in 7 out of 9 evaluable patients through bRT. The median reduction in post-bRT PET-CT compared to pre-bRT PET-CT was − 7.5 (range, − 49 to + 2) or − 42% (range − 96% to + 31%) of the initial SUV (Table 3). The local response rate after bRT was notably high, with an ORR inside the radiation field of 82% (9/11). Specifically, all patients (8/8) who received moderately hypofractionated bRT in single doses of 2.5 to 3.0 Gy demonstrated a local response as defined in decrease in SUV.

Toxicity assessments following bRT demonstrated acute toxicity according to CTCAE grades 1 or 2 in 8 out of 11 patients (73%) (Table 4). No grade 3 toxicities occurred.

**Table 4:** Radiation induced toxicities

	CTCAE-Grade, n (%)		
Acute toxicity (no grade 3 + occurred)	1 + 2	1	2
Fatigue	4 (36)	3 (27)	1 (9)
Radiodermatitis	2 (18)	2 (18)	0 (0)
Nausea	2 (18)	2 (18)	0 (0)
Weight loss	2 (18)	1 (9)	1 (9)
Pain	2 (18)	1 (9)	1 (9)
Local mucositis	1 (9)	1 (9)	0 (0)
Lymphedema	1 (9)	1 (9)	0 (0)
Gastrointestinal disorders (e.g. diarrhea)	0 (0)	0 (0)	0 (0)
Peripheral neuropathy	0 (0)	0 (0)	0 (0)
Any toxicity	8 (73)	8 (73)	2 (18)

*CTCAE* common terminology criteria for adverse events

No significant difference in survival was noted when bRT and systemic bridging therapy was compared (data not shown).

### Response to tisa-cel infusion

**Fig. 1:**
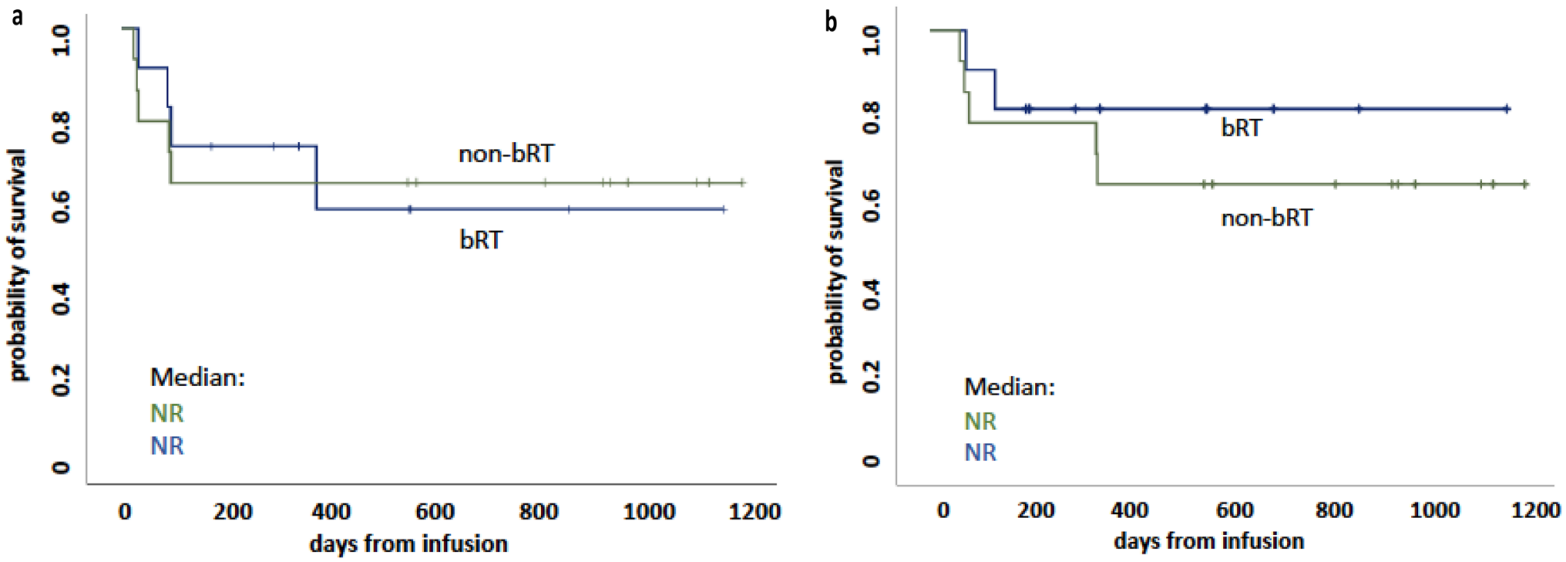
Kaplan–Meier estimates for progression-free survival (**A**) and overall survival (**B**) of patients who received bridging therapy with radiation (bRT) and without radiation (non-bRT)

**Fig. 2:**
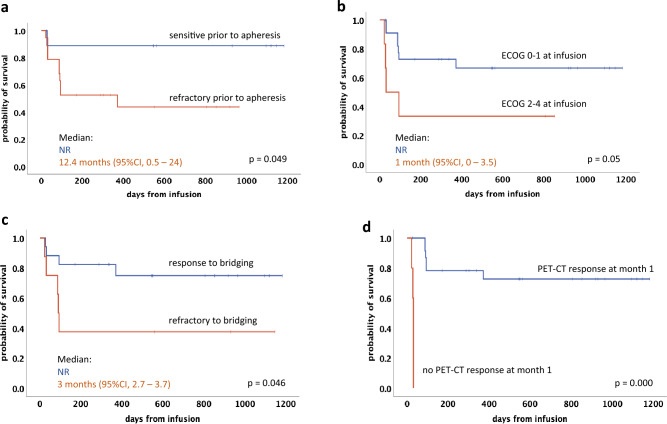
Progression-free survival according to response to chemotherapy prior to apheresis (**A**), ECOG PS (**B**), response to bridging (**C**), PET-CT 1 month after CAR-T (**D**)

**Fig. 3:**
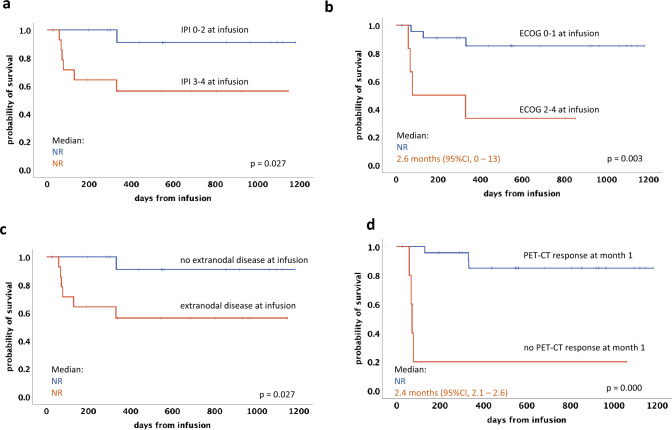
Overall survival according to IPI score at CAR-T infusion (**A**), ECOG PS (**B**), presence of extranodal involvement (**C**), PET-CT 1 month after CAR-T (**D**)

ORR at one month following tisa-cel infusion was 83% (24 patients), with 55% achieving a CR and 28% a PR (Table 5). Early death within 3 months after infusion was reported in four patients, all attributed to progressive disease. Among the responding patients (CR or PR) assessed at month 3 (n = 19), only one patient experienced relapse after achieving a CR at month 3 during long-term follow-up. With a median follow-up of 11.1 months for PFS and of 17.9 months for OS, Kaplan–Meier-estimated rates for the whole cohort at 12 months were 60% for PFS and 74% for OS, respectively (Table 5). When comparing PFS (Fig. 1A) and OS (Fig. 1B) of patients who received bridging therapy with or without radiation, no significant differences were observed. Using log-rank tests, significant predictors of an adverse PFS were identified by univariate analysis (refractory disease status prior to leukapheresis, ECOG PS ≥ 2, non-response to bridging and non-response at month 1 PET-CT) (Fig. 2A, B, C and D). No significant impact on PFS was observed for IPI ≥ 3 (p = 0.21) and extranodal disease (p = 0.21). An inferior OS was statistically associated with an IPI ≥ 3, PS ≥ 2, extranodal disease and non-response at month 1 PET-CT (Fig. 3A–D). No significant impact on OS was seen for refractory disease prior to leukapheresis (p = 0.22) and non-response to bridging (p = 0.09). No impact on PFS or OS was noted for LDH levels at LD (p = 0.09 or p = 0.11, respectively) and prior auto-HCT (p = 0.3 or p = 0.99, respectively).

**Table 5:** Outcome and survival data (total cohort)

ORR all @ day 30 *, n (%)	24 (83%)	p-value
CR @ day 30 PR @ day 30	16 (55%) 8 (28%)	
1-year PFS all (median f/u)	60% (11.1 months)	
1-year OS all (median (f/u)	74% (17.9 months)	
1-year PFS		p = 0.021
CR/PR @ LD	78%	
SD/PD @ LD	40%	
1-year OS		p = 0.195
CR/PR @ LD	82%	
SD/PD @ LD	58%	

*ORR* overall response rate; *PFS* progression-free survival; *OS* overall survival; f/u, follow-up; *LD* lymphodepletion

^*^assessed by PET-CT scan

### Toxicity to tisa-cel infusion

CRS of any grade was observed in 27 patients (93%) patients, with CRS grade ≥ 3 in 10 patients (34%) (Table 6). In the univariate analysis, patients who had a response at month 3 showed a significantly higher incidence of Grade ≥ 3 CRS compared to those who did not respond (p = 0.044). No difference was observed when a multivariate analysis was performed (data not shown). Grade 1 ICANS was reported in 5 patients (17%), while no higher grade was identified.

**Table 6:** Toxicities from CAR-T therapy

	all	PR/CR at month 3	No response at month 3	p-value
CRS all grades, n ≥ 3, n	27 10	17 9	10 1	p = 0.044
ICANS all grades, n ≥ 3, n	5 0	4 0	1 0	n.s
Tocilizumab, n	14	11	3	n.a
Steroids, n	1	1	0	n.a
ICU required, n	2	2	0	n.a
NRM, n	0			n.a

*CRS* cytokine release syndrome; *ICANS* immune effector cell-associated neurotoxicity syndrome; *ICU* intensive care unit; *NRM* non-relapse mortality; *n.a.* not applicable

Hematological toxicity was reported in 21 patients (72%) overall, primarily characterized by severe neutropenia (absolute neutrophil count, ANC < 500 cells/µl) observed in all patients who experienced hematologic toxicity (n = 21) (Table 7). According to the clinical phenotypes of recovery defined by Rejeski et al., 6 patients (29%) showed rapid neutrophil recovery without a second dip, 5 patients (24%) experienced intermittent recovery followed by a second dip below ANC < 0.5/nl after day 21, 10 patients (47%) exhibited continuous severe neutropenia beyond day 21. Prolonged neutropenia persisted in 19 patients (66%), with a median time to neutrophil recovery of 58 days (range, 22–774). It persisted in 13 patients (45%) at month one, 9 patients (30%) at month 3, and 4 patients (20%) at month 6. Two patients continued to experience prolonged neutropenia induced beyond months 9 and 12, respectively. Prolonged severe thrombocytopenia (< 20/nL or requiring transfusion for ≥ 21 days) was observed in 10 patients (34%), with a median time to platelet recovery of 74 days (range, 39–790). Prolonged severe anemia (hemoglobin < 8 g/dL or requiring transfusion) was noted in 13 patients (45%), with a median time to hemoglobin recovery of 56 days (range, 23–776).

**Table 7:** CAR-T cell mediated hematotoxicity

	all	Prolonged at month 1	Prolonged at month 3	Prolonged at month 6	Prolonged at month 9	Prolonged at month 12
Severe neutropenia* n (%)	21/29 (75)	–	–	–	–	–
Prolonged neutropenia** n (%)	19/29 (66)	13/29 (45)	9/23 (39)	4/20 (20)	2/20 (10)	2/13 (15)
Prolonged severe thrombo-cytopenia*** n (%)	10/29 (34)	10/29 (34)	7/23 (30)	3/21 (14)	1/20 (5)	1/14 (7)
Prolonged severe anemia**** n (%)	13/29 (45)	9/29 (31)	7/23 (30)	3/21 (14)	1/20 (5)	1/14 (7)

^*^ANC < 500 cells/µl; **ANC < 1.000 cells/µl and/or G-CSF dependent ≥ 21 days after CAR-T infusion and prolonged ≥ 21 days; *** platelet counts < 20 g/L and/or requiring transfusions ≥ 21 days after CAR-T infusion and prolonged ≥ 21 days; **** hemoglobin < 8 g/dL mmol/l and/or requiring transfusions ≥ 21 days after CAR-T infusion and prolonged ≥ 21 days

Median hospitalization time following CAR-T cell therapy was 22 (range, 18–39) days. No non-relapse deaths occurred during the follow-up period.

## Discussion

This study demonstrates a single-institution real-world experience of CAR-T cell therapy in rr LBCL patients and underscores several pivotal observations. Given the monocentric nature of this analysis, a remarkably consistent and congruous treatment framework is delineated. This pertains to a homogeneous cohort of patients, all of whom received the CAR-T product tisa-cel within the scope of its approval for rr LBCL. Notably this study reports an exceptionally favorable treatment response, characterized by a high incidence of sustained remissions. The study distinguishes itself through a progression-free survival of 60% and an overall survival of 74% at 12 months, markedly surpassing outcomes for tisa-cel and other CD19-directed CAR-T products observed in pivotal trials and other real-world reports. One major aspect of this investigation centers on the significance of a successful bridging strategy as a critical determinant for durable efficacy of CAR-T cell therapy. In this study cohort, 44% of patients received bRT and most of these were chemorefractory prior to leukapheresis. For these patients, the implementation of bRT offers a meaningful option for tumor reduction without the risk of exposing them to clinically significant toxicity. A crucial observation is that all cases of progression in patients receiving bRT occurred beyond the radiation field. This highlights the potential benefit of bRT for patients with bulky disease or those who remain PET-positive in limited sites following bridging but before undergoing lymphodepletion.

To date, various studies have provided data on long-term outcomes of patients after CAR-T cell therapy for rr LBCL, including subsets of patients exhibiting sustained responses beyond 2 years post CAR-T infusion without the need for additional consolidative treatment. (Schuster et al. 2021; Locke et al. 2022; Cappell et al. 2020; Chong et al. 2021). The pivotal single-arm clinical trials conducted in rr LBCL patients who received at least two prior lines of systemic therapy, ZUMA-1 (axi-cel), JULIET (tisa-cel), TRANSCEND (liso-cel) that led to the approval of their respective CD19 CAR-T products showed ORR of 74%, 53% and 63% respectively (Schuster et al. 2021; Neelapu et al. 2023; Abramson et al. 2024). Progression-free survival rates at 12 months for ZUMA-1, JULIET and TRANSCEND were reported as 43% (95% CI, 33–52), not available, and 44% (95% CI, 37–51), respectively. Recognizing the limitations of non-randomized studies, it became imperative to gather real-world data regarding toxicities and treatment responses. A German real-world cohort comprising 356 patients registered in the German Registry for Stem Cell Transplantation (DRST) who received either axi-cel (n = 173) or tisa-cel (n = 183), reported findings consistent with those in the pivotal clinical trials. In alignment with the data presented here, a successful bridging regimen appeared to be favorable for long-term success. Additional risk factors present at the time of CAR-T infusion and identified by univariate analysis to contribute to an unfavorable outcome in our study were ECOG PS ≥ 2, high IPI and extranodal disease. These findings align with other reports that have similarly identified these risk factors (Vercellino et al. 2020; Beyar Katz et al. 2023). An early relapse at month 1 following CAR-T infusion detected by PET-CT was associated with a very poor prognosis whereas a complete metabolic remission at month 3 was linked to a favorable survival outcome. These findings, consistent with others, provide a rationale for early PET-CT to predict responses and in the event of progression or relapse, alternative treatments should be applied (Kuhnl et al. 2022; Georgi et al. 2023).

Divergent and at times conflicting data exist regarding the benefits of bridging therapy. It is therefore imperative to distinguish the characteristics of patients who undergo bridging therapy from those who do not. Several reports reveal the necessity of bridging therapy as a risk factor, as these patients often present with actively progressive disease and a more urgent need for therapy (Pinnix et al. 2020; Nastoupil et al. 2020). A study conducted by the US Lymphoma CAR-T Consortium showed that patients who received bridging therapy showed a trend toward inferior PFS compared to non-bridged patients (p = 0.06). A small subset of patients (n = 11) received RT as a bridging strategy and demonstrated improved PFS compared to those receiving systemic therapy, despite similar baseline characteristics (median PFS 8.9 months (95% CI, 8.2–9.5 months) versus 4.7 months (95% CI, 3.0–6.3 months), p = 0.05, respectively). With the introduction of commercial CAR-T cell therapy, initial studies on the significance of radiotherapy as a bridging strategy were published. Despite the small size of these patient cohorts, the bRT approach appears to exert a favorable effect on long-term outcomes, particularly in chemotherapy-refractory patients. RT is known to impair the capacity of tumor cells to undergo division and proliferation by inducing direct DNA damage, even in chemoresistant lymphomas (Martens et al. 2006; Twyman-Saint Victor et al. 2015). In addition, RT stimulates the migration of pro-inflammatory immune cells towards the irradiated sites, demonstrating the potential to sensitize tumor cells to immunotherapy and induce an abscopal-like effect if combined with CAR-T cell therapy (DeSelm et al. 2018; Weiss et al. 2018; Smith et al. 2019). A recently reported study investigating the patterns of CAR-T failure revealed that the majority of progressions after CAR-T occurred at pre-existing tumor sites (Saifi et al. 2022). This suggests a promising role for bRT for localized disease. In contrast to other reports, the study presented here distinguishes itself through its homogeneous irradiation protocol, which may serve as a model for future clinical trials. The median EQD2α/β = 10 was 31.3 Gy with a narrow range of 31.3–36.0. Despite considerable target volumes, the incidence of radiogenic toxicities remained low, with no grade 3 toxicities. These findings support the suitability of RT as a bridging modality, especially in patients with extensive and bulky tumor involvement. The definition of target volume as involved site RT and the utilization of a relatively low total dose may have contributed to the favorable tolerability of combined treatment approach in our cohort of chemotherapy-refractory patients. All patients who underwent hypofractionated RT (single doses of 2.5–3.0 Gy) exhibited a local response. Although a significant difference in survival was not detected when comparing patients who received bRT and chemotherapy-based bridging therapy, it should be noted that almost all bRT patients (10 out of 11) were refractory to chemotherapy. In summary, our findings suggest that this survival disadvantage of chemorefractory-status can be overcome by bRT.

In a recent report, Roddie et al. have demonstrated superior ORR and survival rates for patients receiving bRT, although statistical significance was not reached (Roddie et al. 2023). The study also revealed that a response to bridging therapy corresponds to a 42% risk reduction in progression or death following CAR-T therapy. Additionally, the authors observed that, particularly for tisa-cel, the addition of the antibody–drug conjugate polatuzumab exhibited twofold effectiveness compared to other chemotherapy-based regimens (Roddie et al. 2023). These findings support the approach of combining bRT with the administration of polatuzumab as implemented in 5 patients in the present study. While data on concurrent treatment with RT and the antibody–drug conjugate polatuzumab is scarce, it can be hypothesized that synergistic effects, particularly concerning cell arrest within the G2/M phase, may contribute to lymphoma cell death (Perrone et al. 2023). Another important observation was the well-tolerated treatment with concurrent polatuzumab and RT.

The primary limitation of our study lies in its small sample size. Nevertheless, given the homogeneous patient cohort in terms of disease entity and CAR-T product applied, important conclusions can be drawn. Although the patient cohort was not selected, as evidenced by the patient characteristics, the results were highly favorable which strengthens the argument that an effective bridging therapy in different types of patients is a key factor for long-term survival. The selection of bridging therapy should be approached judiciously, considering the efficacy and toxicity of prior treatment regimens. A potential strategy may involve contemplating bRT, potentially in combination with antibody- and/or immunotherapy (e.g. polatuzumab), especially for patients with localized disease and those with pre-existing risk factors such as chemorefratoriness, bulky disease, and extranodal involvement. Larger prospective studies are required to adequately evaluate the impact of bRT and potentially a combination of radiation and immunotherapy as a bridging modality before CAR-T infusion. Furthermore, systematically designed clinical trials are warranted to address questions regarding the localization, optimal dosage, and timing of bRT (Hovhannisyan et al. 2023).

### Supplementary Information

Below is the link to the electronic supplementary material.

Supplementary file1 (DOCX 25 KB)

## Data Availability

Patient records of University Hospital Jena.
