# Supplementary material for: Effective bridging strategies prior to infusion with tisagenlecleucel results in high response rates and long-term remission in relapsed/refractory large B-cell lymphoma: findings from a German monocentric study
Source: J Cancer Res Clin Oncol. 2024 May 1;150(5):224. doi: 10.1007/s00432-024-05765-8 (PMC11062962; doi:10.1007/s00432-024-05765-8)
Supplement: Supplementary file 1 — Supplementary file1 (DOCX 25 KB) [file 432_2024_5765_MOESM1_ESM.docx]

**Table S1: Characteristics of bridged patients (non-RT vs bRT)**

|  | non-RT bridging | bRT |
| --- | --- | --- |
| Patients infused | 14 | 11 |
| Median age, years (range) | 62.5 (34-74) | 63 (50-73) |
| Female, n, (%) | 5 (36) | 5 (45) |
| LDH @ Leukapheresis |  |  |
| LDH ≤ ULN, n (%) | 7 (50) | 6 (55) |
| LDH > ULN, n (%) | 7 (50) | 5 (45) |
| LDH @ LD |  |  |
| LDH ≤ ULN, n (%) | 8 (57) | 8 (73) |
| LDH > ULN, n (%) | 6 (43) | 3 (27) |
| Prior auto-HCT, n (%) | 8 (57) | 3 (27) |
| ≥ 3 treatment lines @ apheresis, n (%) | 14 (100) | 6 (55) |
| Interval leukapheresis to CAR-T infusion (days), median (range) |  |  |
| Response to last treatment prior apheresis |  |  |
| CR/PR, n, (%) | 6 (43) | 2 (18) |
| SD/PD, n (%) | 7 (50) | 9 (82) |
| n.d., n (%) | 1 (7) |  |
| Refractory to any line prior apheresis, n (%) | 7 (50) | 9 (82) |

**Abbreviations:** RT, radiotherapy; bRT, bridging therapy including radiation; LDH, lactate dehydrogenase; LD, lymphodepletion (lymphodepleting chemotherapy); ULN, upper limit normal; IPI international prognostic index; auto-HCT, autologous hematopoietic cell transplantation; CR, complete remission; partial remission; SD, stable disease; PD, progressive disease; n.d., no data

**Table S2: Description of lines of therapy prior to leukapheresis**

| Patient No | 1^st^ line | 2^nd^ line | 3^rd^ line | 4^th^ line | 5^th^ line |
| --- | --- | --- | --- | --- | --- |
| 1 | R-CHOP (6x)  refractory | Radiation  refractory | R-DHAP (2x)  refractory | R-ICE (2x)  refractory | Radiation  refractory |
| 2 | R-CHOP (6x)  response | R-DHAP (3x)  response | auto-HCT  response | Radiation  response | R-CHOP  response |
| 3 | R-CHOP (6x)  response | Radiation  response | (R-DHAP 4x)  response | auto-HCT  response |  |
| 4 | R-CHOP (6x)  response | R-DHAP (2x)  response | auto-HCT  response |  |  |
| 5 | R-CHOP (6x)  response | R-DHAP (3x)  response | auto-HCT  response |  |  |
| 6 | R-CHOP (4x)  response | radiation (2x)  response | R-DHAOx (6x)  response | R-ICE (1x)  response |  |
| 7 | R-CHOP (4x)  refractory | R-DHAP (3x)  refractory | R-ICE (1x)  refractory |  |  |
| 8 | R-CHOP (6x)  response | R-DHAP (3x)  refractory | auto-HCT  refractory |  |  |
| 9 | R-CHOP (6x)  refractory | R-DHAP (2x)  refractory | R-ICE (2x)  response |  |  |
| 10 | R-CHOP (6x)  response | R-Bendamustine (6x)  refractory | R-Polatuzumab (6x)  refractory | Radiation  response |  |
| 11 | R-CHOP (3x)  refractory | R-DHAP (2x)  refractory |  |  |  |
| 12 | R-CHOP (6x)  refractory | R-DHAP (2x)  refractory |  |  |  |
| 13 | R-CHOP (6x)  response | R-DHAP (1x)  refractory | MATRIX-induction (3x)  refractory | auto-HCT  refractory |  |
| 14 | R-CHOP (6x)  response | R-DHAP (3x)  response | auto-HCT  response |  |  |
| 15 | R-CHOP (6x)  refractory | R-DHAP (2x)  refractory |  |  |  |
| 16 | R-CHOP (8x)  response | R-MTX (1x)  response | R-DHAP (3x)  response | auto-HCT  response |  |
| 17 | R-CHOP (5x)  response | R-Pola-ICE (2x)  refractory |  |  |  |
| 18 | R-CHOP (6x)  response | R-Gem-Ox (1)  response | R-DHAOx (3x)  response | auto-HCT  response |  |
| 19 | R-CHOP (4x)  refractory | R-DHAP (1x)  refractory | R-ICE (2x)  refractory |  |  |
| 20 | R-CHOP (6x)  refractory | R-DHAOx (3x)  refractory |  |  |  |
| 21 | R-CHOP (6x)  response | R-MTX (1x)  response | R-Cytarabin-Thiotepa (2x)  response | auto-HCT  response |  |
| 22 | R-CHOP (6x)  response | R-DHAP (3x)  refractory | R-Pola-Bendamustine  Refractory | Radiation  response |  |
| 23 | R-CHOP (6x) + 2x HD-MTX  response | R-MTX-HDAraC (3x)  refractory | Whole brain radiation  response |  |  |
| 24 | R-CHOP (5x)  refractory | R-DHAP (2x)  refractory | R-ICE (3x)  refractory | auto-HCT  refractory |  |
| 25 | R-CHOP (6x) + 2x HD-MTX  response | R-DHAP (2x)  refractory | R-Pola-Bendamustine  refractory |  |  |
| 26 | R-CHOP (7x)  refractory | R-Pola-ICE (4x)  refractory |  |  |  |
| 27 | R-CHOEP (6x)  refractory | R-DHAP (4x)  refractory | R-Pola  (2x)  refractory |  |  |
| 28 | R-CHOP (6x)  refractory | R-ICE (2x)  response |  |  |  |
| 29 | R-CHOEP (4x)  response | R-DHAP (3x)  response | auto-HCT  response | R-ICE (2x)  response |  |

**Abbreviations:** No, number; R-CHO(E)P, Rituximab/Cyclophosphamide/Doxorubicin Hydrochloride/(Etoposide)/Prednisone; R-ICE, Rituximab/Ifosfamide/Carboplatin/Etoposide; R-DHAP, Rituximab/Dexamethasone/Cytarabine/Cisplatin; R-DHAOx, Rituximab/Dexamethasone/Cytarabine/Oxaliplatin; HD-MTX, high-dose Methotrexate; MATRIX-induction, Rituximab/high-dose Methotrexate/Cytarabine/Thiotepa; auto-HCT, autologous hematopoietic cell transplantation

**Table S3: Location of irradiated tumor sites**

| **Target site** | **n (%)** |
| --- | --- |
| Paraaortic | 6 (55) |
| Renal pelvis (left) | 3 (27) |
| Common iliac, external iliac, inguinal | 3 (27) each |
| Lung (left lower lobe) | 2 (18) |
| Thigh, lower leg, mediastinum, mesenterium, orbita | 1 (9) each |
